# Consensus formalisé: recommandations de pratiques cliniques pour la prise en charge de la lombalgie aiguë du patient africain

**DOI:** 10.11604/pamj.2015.22.240.8120

**Published:** 2015-11-13

**Authors:** Mohamed Elleuch, Abdellah El Maghraoui, Brahim Griene, Mati Nejmi, Souhaibou Ndongo, Alain Serrie

**Affiliations:** 1 Hôpital La Rabta, Tunis, Tunisie; 2 Hôpital Militaire d'Instruction Mohammed V, Rabat, Maroc; 3 Centre Pierre et Marie Curie d'Alger, Algérie; 4 Hôpital Cheikh Khalifa Bin Zayed, Casablanca, Maroc; 5 Université Cheikh Anta Diop de Dakar, Sénégal; 6 Hôpital Lariboisière de Paris, France

**Keywords:** Lombalgie aigüe, Afrique, Recommandation, acute lowback pain, Africa, Recommendation

## Abstract

La lombalgie aiguë est la pathologie rhumatismale la plus fréquente en Afrique. L’épidémiologie et la présentation clinique ne diffèrent pas de celles observées sur les autres continents. En revanche, les aspects psycho-sociaux, la disponibilité des traitements, l'accès aux soins et le poids culturel des médecines traditionnelles sont autant de spécificités qui ont conduit à la réalisation du 1er consensus d'experts en rhumatologie pour la prise en charge du patient africain. Destiné aux praticiens, ce travail collaboratif multinational a pour objectif de fournir 11 recommandations de pratiques cliniques simples, fondées sur les preuves, et adaptées aux conditions de l'exercice médical en Afrique. Leur ambition est d'améliorer la prise en charge de la lombalgie aigue par une évaluation initiale clinique pertinente, une diminution des examens radiologiques inutiles, une prescription médicamenteuse adéquate et l'abandon de procédures invasives inappropriées.

## Introduction

La lombalgie est une douleur ou gêne fonctionnelle située entre la douzième côte et le pli fessier, associée ou non à des irradiations dans les membres inférieurs. Elle est typiquement classifiée selon sa durée: les six premières semaines correspondent à la période de lombalgie aiguë, entre six et douze semaines la lombalgie est dite subaiguë, et au-delà, la lombalgie est dite chronique ou persistante [1]. Plus d'une trentaine de recommandations relatives au diagnostic et à la prise en charge des lombalgies ont été publiées au cours des 20 dernières années [2, 3]. Elles sont globalement concordantes, illustrant l′absence de nouveaux éléments de preuve montrant de meilleurs résultats avec de nouvelles approches diagnostiques et thérapeutiques et/ou de nouvelles preuves montrant l′inefficacité des interventions existantes. La diffusion des recommandations cliniques et leur application par les professionnels de la santé demeure un problème significatif. Comprendre pourquoi les médecins ne les suivent pas est un prérequis pour améliorer cette situation. En effet, la multiplicité des recommandations et le manque de temps sont les facteurs principaux dans leur non application. Une revue des travaux scientifiques réalisés ces 20 dernières années permet de faire une mise au point et d'améliorer l'implémentation de ces recommandations.

## Méthodes

Ces recommandations ont été’ élaborées par la méthode du consensus formalise’, de type Delphi-modifié, décrite par la Haute Autorité de Santé (HAS) française [4]. Elle a reposé, d'une part, sur l'analyse et la synthèse critiques de la littérature médicale disponible (PubMed), et, d'autre part, sur l'avis et les données non publiées d'un groupe de 16 rhumatologues pratiquant sur le continent africain qui a réalisé une cotation en deux tours des propositions de recommandations établies par le groupe de pilotage.

### Etat actuel des connaissances

### La démarche diagnostique

#### Recommandation #1 - Accord professionnel fort

**Devant une lombalgie aiguë, l’évaluation de la douleur doit être accompagnée d'un examen clinique complet et bien conduit à la recherche de signes de gravité (‘red flags’) évoquant une étiologie grave sous-jacente ou un risque de complications**

Dans les pays occidentaux, environ 70 à 85% des adultes souffrent au moins une fois au cours de leur vie d'un épisode de lombalgie. Pour la plupart, le premier épisode survient entre 20 et 40 ans et constitue ainsi la première raison de consulter à un âge adulte. La prévalence annuelle se situe autour de 30% et la prévalence ponctuelle autour de 20% [5]. Cette différence reflète probablement la nature instable et épisodique de la lombalgie. Par ailleurs, la prévalence est variable en fonction de l’âge avec un maximum entre 40 et 60 ans. Les facteurs de risque de la lombalgie aiguë les plus fréquemment cités dans la littérature sont en rapport avec une mauvaise hygiène posturale. Ainsi, le port, le levage, le tirage et la poussée de charges lourdes, la flexion du tronc de plus de 60°, les mouvements de torsion, le travail répétitif, les positions statiques et les vibrations sont les plus retrouvés. La douleur survient brutalement, en général après un effort brusque ou inhabituel. Elle entraîne une contracture reflexe des muscles du dos, qui bloquent les mouvements à ce niveau douloureux. Le lumbago (trouble musculo-squelettique) est la cause la plus fréquente de lombalgie aigue: il dure en général quelques jours, mais tend à récidiver dans 1/3 des cas. Ces douleurs récurrentes peuvent persister au cours des deux premières années, avec des douleurs modérées à sévères respectivement de 15 à 30% [6]. Dans les pays africains, l’épidémiologie semble tout à fait comparable, même si les études sont rares [7, 8]. Les lombalgies représentent ainsi la première cause de consultation en rhumatologie et un des motifs les plus fréquents de consultation en médecine générale [9]. Généralement, on distingue deux types de lombalgies. Les lombalgies spécifiques (ou symptomatiques) présentent des symptômes clairement identifiés (infection, tumeur, fracture, etc.). A l'inverse, les lombalgies non spécifiques (ou communes) sont en rapport avec des lésions dégénératives bénignes du rachis ou n'ont pas de cause identifiable précise. Elles représentent 85 à 90% des cas [10, 11]. Une approche pratique d’évaluation est de se focaliser sur l'histoire de la maladie et de procéder à un examen clinique pour préciser ou non la probabilité d'une pathologie sous-jacente et de mesurer la présence et le niveau d'une participation neurologique [12]. Il est aussi important de noter s'il s'agit d'un premier épisode ou récurrent. Les épisodes récurrents sont en général plus douloureux. Cette approche permet une classification pertinente des patients et améliore la prise en charge en fonction des données retrouvées. Pour affiner le diagnostic et la prise en charge initiale, il est essentiel de rechercher les signes d'alerte (‘red flags’) d'une éventuelle cause symptomatique (Tableau 1) [13, 14]. Ces signes de gravité doivent attirer l'attention du clinicien qui va alors réaliser des examens complémentaires qui lui permettront de poser un diagnostic précis et donc de proposer un traitement adapté. Ils sont utilisés pour faire la distinction entre un épisode commun bénin et un problème plus important qui nécessite une prise en charge et un traitement urgent.

**Tableau 1:** Les signes d'alerte ou ‘red flags’

**Age > 55 ans**
**Traumatisme violent récent chez un jeune ou plus ou moins d'ostéoporose chez un sujet âgé**
**Douleur constante, progressive et non mécanique**
**Infection, douleur sévère et antécédent de chirurgie lombaire**
**Antécédents de tumeur maligne, métastases osseuses, perte de poids inexpliquée, douleur nocturne, rétention urinaire**
**Corticothérapie au long cours**
**Toxicomanie IV, immunodéficience, VIH**
**Maladie systémique**
**Pathologie intra-abdominale majeure**
**Déficit neurologique, syndrome de la queue de cheval, rétention urinaire**
**Déformation structurelle**
**Fièvre, utilisation récente d'antibiotiques ou d'anti-infectieux**

#### Recommandation #2 - Accord professionnel fort

**Les facteurs de risque de chronicisation (‘yellow flags’), notamment les croyances, les aspects sociaux et environnementaux du patient africain, doivent être répertoriés dès le début de la prise en charge**.

Environ 2 à 7% des patients lombalgiques passeront à la chronicité [1], dont les facteurs de passage sont essentiellement psychologiques mais également environnementaux et sociaux [15]. Ces indicateurs doivent être recherchés et limités lorsque cela est possible dès la phase de prise en charge initiale. Les facteurs psychosociaux augmentent le risque de développer, ou de perpétuer une lombalgie chronique et une invalidité à long terme, y compris la perte d'emploi. Qualifiés de « yellow flags » ou « drapeaux jaunes », ces facteurs de risque de chronicisation peuvent revêtir de nombreuses formes (Tableau 2) [16–18]. Les principaux indicateurs de risque de passage à la chronicité sont représentés par un état dépressif, l'isolement, la peur de se faire mal, une insatisfaction au travail, des tâches physiques lourdes, un faible soutien social, du stress et une douleur intense. Il est nécessaire d'agir sur ces éléments lorsque cela est possible. Les facteurs émotionnels et la détresse émotionnelle doivent être évalués car ils sont d'aussi forts prédicteurs des résultats de la prise en charge de la lombalgie aigue que ceux de l'examen physique ou la sévérité et la durée de la douleur. L’évaluation des facteurs psychosociaux permet d'identifier les patients susceptibles d'avoir une récupération retardée et nécessitant une aide ciblée. Une récente publication révèle que l'association de comorbidités à la lombalgie, notamment cardiovasculaires, a un impact plus important sur l'exercice professionnel que le mal de dos isolé. Ainsi, la recherche de comorbidités doit, elle aussi, faire partie des facteurs pronostiques à rechercher [19]. Dans certains pays d'Afrique, il n'est pas rare que les maladies rhumatismales soient assimilées a’ un mal d′origine métaphysique tel que le mécontentement d'un ancêtre défunt, un sort jeté’ par des esprits maléfiques, un ensorcellement ou un envoutement. Cette conception ne semble pas influencée par le niveau d′instruction et s′observe aussi chez les patients ayant un niveau d′instruction élevé’. La prise en compte de ces considérations est d'un grand intérêt dans la mesure ou’ elle fait partie intégrante de la prise en charge globale du patient d'une part, et, d'autre part, elle explique souvent le recours à des médecines traditionnelles dont il faut comprendre l'importance pour rendre la stratégie thérapeutique ‘rationnelle’ plus acceptable par le patient.

**Tableau 2:** Les facteurs de risque de chronicisation de la maladie ou ‘yellow flags’

Nature	Exemples
**Croyances, représentations et jugements**	Croyances concernant la douleur: intervention surnaturelle, douleur incontrôlable, s'aggravant inéluctablement. Faible espoir de guérison et de reprise du travail
**Réponses émotionnelles**	Détresse psychologique Posture de retrait social Craintes, stress Anxiété
**Comportements face à la douleur (y compris les stratégies d'adaptation)**	Évitement d'activités ou niveau d'activité réduit en raison des craintes de majoration de la douleur Dépendance excessive aux traitements passifs (repos au lit, analgésiques)

### Les examens radiologiques

#### Recommandation #3 - Accord professionnel fort

**En présence d'une lombalgie aiguë sans drapeaux rouges, il n'y a pas d'indication à la demande de radiographies standard, ni d'autres examens complémentaires dans les 7 premières semaines d’évolution**.

Une revue systématique de 31 études sur l′association entre les résultats des radiographies de la colonne lombaire et les lombalgies a montré que la dégénérescence, définie par la présence d′un pincement discal, des ostéophytes et une condensation des plateaux est toujours associée positivement à la douleur lombaire avec un Odd Ratio de 1,2 (IC 95% 0,7 à 2,2) à 3,3 (IC 95% 1,8 à 6,0). La présence de spondylolyse/listhésis, de spina bifida, d'anomalies de transition, de signes d'arthrose et de la maladie de Scheuermann ne semble pas être associée à la douleur lombaire (niveau de preuves I). Il n′existe aucune preuve sur l′association entre signes dégénératifs à la phase aiguë et la transition vers des lombalgies chroniques [20]. Une revue récente de la littérature en radiologie diagnostique (imagerie par résonance magnétique, scintigraphie, tomodensitométrie, radiographies) a conclu que, pour des adultes de moins de 50 ans ne présentant aucun signe ou symptômes de maladie systémique, l′imagerie ne permet pas d′améliorer le traitement de la lombalgie. Pour les patients de 50 ans et plus, ou de ceux dont des symptômes suggèrent une maladie systémique, la radiographie standard et la recherche de stigmates biologiques de l'inflammation peuvent presque complètement exclure des maladies sous-jacentes. Les auteurs ont conclu que les examens d′imagerie devraient être réservés aux patients chez qui une chirurgie est envisagée ou ceux chez qui une maladie systémique est fortement soupçonnée (niveau de preuves I) [21, 22]. Aucune recommandation publiée ne préconise la prescription d'examens d'imagerie en cas de lumbago [23, 24]. La découverte d'un nombre important d'anomalies vertébrales, discales ou des articulaires postérieures chez des sujets indemnes de lombalgie a permis de conclure que ces images a priori pathologiques découvertes au cours des douleurs lombaires pouvaient tout à fait être une coïncidence. Seuls des éléments de présomption pourront être avancés, tout en gardant à l'esprit l'extrême banalité des remaniements dégénératifs du rachis lombaire qui restent somme toute souvent asymptomatiques. Ainsi, en présence d'une lombalgie aiguë sans drapeaux rouges, il n'y a pas d'indication à l'exécution de radiographies standards car la cause est habituellement spontanément résolutive et l'imagerie ne modifie pas la stratégie thérapeutique. Une irradiation non nécessaire devrait être évitée. Enfin, l'imagerie a un haut potentiel irradiant et, dans le contexte de faibles ressources, elle n'est pas sans impact économique. La radiologie de routine est par contre recommandée pour l’évaluation initiale d'une possible compression vertébrale liée à une fracture chez les patients à hauts risques tels que ceux évoquant une histoire d'ostéoporose, de cancer ou d'utilisation prolongée de corticoïdes. En effet, les recommandations de la Haute Autorité de Santé (HAS) précisent qu'aucun bilan radiologique n'est utile dans les sept premières semaines d’évolution d'une lombalgie aigue, sauf si le contexte et l'examen clinique ou encore la biologie sont en faveur d'une lombalgie symptomatique (fracture, tumeur, infection) ou d'un syndrome de la queue de cheval. Le délai pourra se voir raccourci si l’évolution sous traitement bien conduit n'est pas favorable dans ce délai. En dehors de ces cas, l'absence d'indication pour un bilan radiologique est généralement motivée par le fait qu'une grande majorité des lombalgies aigues guérissent en moins d'un mois.

### La prise en charge médicamenteuse

#### Recommandation #4 - Accord professionnel fort <

Les antalgiques de paliers 1 sont le traitement de première intention, dans le respect de leurs contre-indications et précautions d'emploi. L'efficacité des anti-inflammatoires non stéroïdiens (AINS) doit être évaluée après quelques jours de traitement. Les formes topiques ne sont pas recommandées sauf lors de massages à visée antalgique.

#### Recommandation # 5 - Accord professionnel fort

**En l'absence de contre-indications, les antalgiques de paliers 2 (opioïdes faibles) associés ou non aux antalgiques de palier 1 sont utilisés en cas d’échec des antalgiques de palier 1, en prévenant les éventuels effets indésirables (constipation, nausées et vomissements, vertiges, chute tensionnelle), notamment à forte dose et/ou chez le sujet âgé. Leur efficacité doit être évaluée après 2 ou 3 jours de traitement**.

De la physiopathologie de la lombalgie aigue, on retient principalement les aspects mécaniques expliquant ainsi en grande partie son évolution et son pronostic qui est le plus souvent favorable. Si une amélioration est possible au cours des premiers jours, on observe généralement un rétablissement dans 90% des cas en 6 semaines. En revanche, dans 2 à 7% des cas, les patients développent une lombalgie chronique. Dans les pays occidentaux, les douleurs chroniques représentent 75% à 85% de l′absentéisme au travail [2, 14]. Les principaux objectifs du traitement de la lombalgie aiguë sont ainsi de soulager la douleur, d'améliorer les capacités fonctionnelles et d’éviter le passage à la chronicité. L'utilisation de thérapeutiques médicamenteuses en Afrique est la conjonction de l’état des connaissances, à travers les publications internationales, et de la disponibilité des produits, sous forme de princeps ou de génériques. Dans le contexte africain, la prise en charge médicamenteuse ne diffère pas de celle des autres continents. En revanche, sa mise en ‘uvre peut être parfois plus compliquée. Le paracétamol est le traitement de première intention dans la majorité des recommandations en raison de sa bonne tolérance, en particulier digestive [14, 25]. La dose recommandée est généralement de 3 g/jour [26]. L'utilisation de cette posologie, en l'absence de contre-indication, ne comporte pas ou peu de risque hépatique en dehors d'une élévation possible des transaminases, ce qui n'est pas le cas de l'administration prolongée. Il convient de noter cependant qu'il n'existe pas d’étude contrôlée contre placebo avec le paracétamol. L'effet antalgique des anti-inflammatoires non stéroïdiens (AINS) a été largement démontré par de nombreux essais randomisés contre placebo, compte tenu des mécanismes qui prévalent, justifiant leur utilisation seul en première intention [27]. Leur intérêt est discutable dès que la lombalgie persiste. D'autres études, également contrôlées et randomisées, montrent qu'ils sont plus efficaces que le paracétamol, mais il est raisonnable de prescrire ce dernier en première intention pour son profil plus sécurisant et son faible prix [14, 25, 28]. Si le bénéfice des AINS est clairement établi, une évaluation de l’état cardiovasculaire, rénal et gastro-intestinal est néanmoins nécessaire avant leur utilisation. Il est important de réévaluer l'efficacité des antalgiques de palier I après 4 à 5 jours de traitement. Si le soulagement n'est pas optimal, une association aux opioïdes faibles sera envisagée. Les antalgiques de palier I peuvent ainsi être prescrits seuls ou en association avec les opioïdes faibles.

#### Recommandation # 6 - Accord professionnel fort

**Les décontracturants musculaires, les antiépileptiques, les antidépresseurs, les anxiolytiques, les corticoïdes par voie générale et les infiltrations épidurales ne sont pas indiqués dans le lumbago**.

Les décontracturants musculaires représentent le traitement pour lequel il y a le plus de contradictions entre les différentes recommandations. Certains le recommandent en troisième intention après les antalgiques et les AINS [14]. S'ils sont plus efficaces que le placebo, ils n'ont pas l'efficacité des AINS et sont susceptibles d'induire des effets indésirables qui compromettent leur rapport bénéfice/risque [26, 28]. De même, de nombreuses autres classes thérapeutiques ont été évaluées dans le traitement de la lombalgie aiguë. Aucune n'a démontré une efficacité suffisante pour justifier leur recommandation dans la prise en charge thérapeutique.

### Les mesures non pharmacologiques

#### Recommandation # 7 - Accord professionnel fort

**Le repos strict au lit ne doit pas être recommandé. Les activités de la vie courante, compatibles avec la douleur, doivent être poursuivies et encouragées, tout en évitant les mauvaises postures**.

La poursuite des activités de la vie courante, compatibles avec la douleur, tout en évitant les mauvaises postures au travail doit être encouragée au cours de la lombalgie aiguë. Le repos au lit doit être évité le plus possible pour éviter la persistance des symptômes [29, 30]. Au contraire, la reprise précoce des activités est source d'amélioration de la symptomatologie aiguë [31, 32]. Une prise en charge initiale adaptée de la douleur doit pouvoir agir sur l'intensité et la durée de la lombalgie, contribuant à réduire ainsi l'incapacité fonctionnelle. De même, les peurs et croyances sur la relation douleur et activité physique doivent être évaluées, et peuvent faire l'objet d'une information voire d'une prise en charge ciblée. Il existe au moins six revues systématiques et 10 essais randomisés contrôlés ayant évalué l′effet du repos au lit pour la lombalgie aiguë [14, 33–35]. En comparant le repos à d′autres traitements, notamment les exercices, la physiothérapie, les manipulations vertébrales, ou les AINS, il a été montré soit qu'il n'y avait pas de différence, soit que le repos au lit donnait de moins bons résultats que ce soit sur la diminution de la douleur, le taux de récupération, le temps nécessaire pour retrouver leurs activités quotidiennes ou la durée des congés de maladie. Cinq études ont constaté que le repos au lit n′améliorait pas l’état du patient ou l'aggravait, comparé à l'abstention thérapeutique ou un placebo. Il semble maintenant y avoir un large consensus s'accordant à déconseiller le repos au lit en tant que traitement des douleurs lombaires. Quelques recommandations stipulent que si le repos au lit est indiqué (en raison de la gravité de la douleur), il ne devrait pas être conseillé pour plus de 2 jours. Les effets indésirables du repos au lit sont l'aggravation de la raideur, l′atrophie musculaire, la perte de densité minérale osseuse, et le risque de thrombose veineuse. L'alitement prolongé peut entraîner une invalidité chronique et peut nuire à la réadaptation [30, 36]. Il n'y a pas de corrélation entre le maintien d'une activité de loisirs et l′intensité de la lombalgie [37, 38]. Les patients souffrant de lombalgie aiguë peuvent ainsi faire en toute sécurité de faibles mouvements de flexion- extension [34, 33]. Une revue systématique de huit essais randomisés contrôlés a constaté que les conseils pour rester actif sont associés à une amélioration plus rapide, une moindre incapacité chronique et une diminution des arrêts de travail par rapport au repos au lit ou par rapport aux soins habituels [35, 39]. Deux études ont démontré des taux plus rapides de reprise, moins de douleur et d′invalidité, et cinq études ont mis en évidence une diminution des congés de maladie et de l'incapacité chronique par rapport au traitement médical classique. Enfin, une revue récente a également montré que le maintien d'une activité physique avait significativement réduit la douleur et amélioré la fonctionnalité à 3 - 4 semaines et à 12 semaines par rapport au repos strict au lit [14, 34].

#### Recommandation # 8 - Accord professionnel relatif

**L'acupuncture est peu pratiquée en Afrique. Elle n'est pas indiquée dans le traitement du lumbago,même si elle pourrait avoir une certaine efficacité du fait d'un effet placebo**.

Dans une mise au point même si elle pourrait avoir une certaine efficacité du fait d'un effet placebo.

Dans une mise au point,Cassazza et al. indiquent qu'aucun avantage substantiel n'a été démontré avec l'acupuncture dans la prise en charge du lumbago [29’> Cassazza et al. indiquent qu'aucun avantage substantiel n'a été démontré avec l'acupuncture dans la prise en charge du lumbago [29]. Comparée aux traitements pharmacologiques, l′acupuncture bénéficie d'une faible incidence d'effets indésirables, mais la preuve de son efficacité reste limitée [40, 41].

#### Recommandation # 9 - Accord professionnel fort

**Dans le contexte africain, les manipulations vertébrales ne sont pas recommandées. Elles pourraient avoir un intérêt dans la prise en charge du lumbago mais nécessitent un personnel qualifié sous peine de voir la situation clinique s'aggraver**.

L'intérêt des manipulations vertébrales dans le traitement du lumbago est controversé dans la littérature, y compris avec un personnel formé et qualifié. D'après Chou et al., les cliniciens devraient envisager un traitement non pharmacologique telle que les manipulations vertébrales chez les patients qui ne présentent pas d'amélioration avec le traitement médicamenteux [25].

### L’éducation et le suivi du patient

#### Recommandation # 10 - Accord professionnel fort

**Il est recommandé d’éduquer le patient sur les facteurs de risque et les causes hypothétiques (information orale et/ou écrite) et de le rassurer sur le bon pronostic de l'affection (amélioration en moins d'un mois)**.

Le milieu ethnique et culturel peut affecter significativement les possibilités d’évaluer et de traiter la douleur aigue du dos, mais ne devraient pas être utilisées comme des stéréotypes. L'un des principaux objectifs de l’éducation est d'augmenter la volonté des patients à s'engager dans des activités qui ont été évitées depuis longtemps. Une revue non systématique a évalué l'efficacité des interventions éducatives pour les lombalgies [42, 43]. Ainsi, la délivrance d′un livret pédagogique est susceptible de réduire le nombre de visites à un médecin généraliste pour lombalgies [44, 45]. Une autre étude a montré qu′une séance pédagogique de 15 minutes avec une infirmière formée, ainsi que la remise d′un livret pédagogique et d'un appel téléphonique de suivi a permis une plus grande satisfaction à court terme et une meilleure connaissance perçue par rapport aux soins habituels [46]. Cependant, les symptômes, l’état fonctionnel et l′utilisation des différentes modalités thérapeutiques n′étaient pas significativement différents [47]. En revanche, une revue Cochrane, réalisée récemment, a montré qu'une séance individuelle de 2,5 heures est aussi efficace qu'une intervention non éducative sur la douleur et la fonction à long terme. Des séances plus courtes, la distribution de livret de conseils sans entretien pédagogique ne semblaient pas efficaces [43].

La plupart des recommandations insistent sur l'importance de rassurer les patients en expliquant que la pathologie n'est pas grave et qu′une reprise rapide des activités peut être prévue [48]. Il est donc important de donner une explication complète dans des termes simples que le patient comprend comme, par exemple: le mal de dos est très fréquent; bien que les récidives soient fréquentes, l’évolution est en général très bonne; son origine pourrait provenir de différentes structures anatomiques, tels que les muscles, les disques, les articulations ou les ligaments.

#### Recommandation # 11 - Accord professionnel fort

**Il est recommandé de réévaluer entre la quatrième et la sixième semaine les patients qui n'ont pas eu d'amélioration suite à leur prise en charge initiale et ceux dont la situation clinique s'est détériorée. Dans ces situations, un avis spécialisé est souhaitable**.

Les nombreuses recommandations internationales sont assez hétérogènes, ne serait-ce qu'en termes de langue, mais aussi de qualité. Néanmoins, on note une grande cohérence en termes de prise en charge qui se résume ainsi: éducation du patient; prise en charge médicamenteuse; exercices physiques et activités quotidiennes; ‘manipulations’ ou autres mesures non pharmacologiques; adressage aux spécialistes, notamment en cas de ‘red flags’. Bien que rarement mentionné, il semble raisonnable pour la plupart des patients de renouveler l'examen clinique dans les premières semaines suivant l’épisode aigu, à moins que le problème soit complètement résolu. Cette échéance peut également s'avérer utile pour asseoir le contrat thérapeutique entre le praticien et son patient.

## Conclusion

Si la pensée médicale occidentale est aujourd'hui dominée par une approche rationnelle des maladies et de leurs traitements, dans certaines régions d'Afrique, la maladie est encore perçue comme la conséquence d'une origine surnaturelle. Les comportements qui en résultent, absolument indissociables à la culture, peuvent parfois être très marqués au cours des affections rhumatismales, dominées, au plan symptomatique, par la douleur et la physiopathologie souvent obscure. De plus, de nombreux patients sont confrontés à des difficultés d'accès aux soins, pour des raisons structurelles, géographiques ou par manque de ressources financières. S'y ajoute le nombre limité de spécialistes. Ainsi, il est nécessaire d'adopter une approche régionale pour développer des stratégies pertinentes susceptibles d'améliorer les comportements de soins et de proposer des stratégies thérapeutiques appropriées au contexte.

## Conflits d'intérêts

Les auteurs ne déclarent aucun conflit d'intérêt.

## Contributions des auteurs

Tous les auteurs ont contribué à la conduite de ce travail. Tous les auteurs déclarent également avoir lu et approuvé la version finale du manuscrit. Ces travaux ont été réalisés avec le soutien institutionnel de Sanofi-Aventis Groupe.
